# Association Between Various Types or Statuses of Smoking and Subjective Cognitive Decline Based on a Community Health Survey of Korean Adults

**DOI:** 10.3389/fneur.2022.810830

**Published:** 2022-04-29

**Authors:** Ji Hee Kim, In Bok Chang, Yoo Hwan Kim, Chan Yang Min, Dae Myoung Yoo, Hyo Geun Choi

**Affiliations:** ^1^Department of Neurosurgery, Hallym University College of Medicine, Anyang, South Korea; ^2^Department of Neurology, Hallym University College of Medicine, Anyang, South Korea; ^3^Hallym Data Science Laboratory, Hallym University College of Medicine, Anyang, South Korea; ^4^Department of Otorhinolaryngology-Head and Neck Surgery, Hallym University College of Medicine, Anyang, South Korea

**Keywords:** cigarette, cognition, dementia, smoking, subjective cognitive decline

## Abstract

**Objectives:**

The relationship between smoking and subjective cognitive decline (SCD), which is defined as the subjective perception of cognitive decline, is not well known. This study aimed to investigate the relationship of various types of smoking, including E-cigarette smoking and the use of E-liquid, with the incidence of SCD among Korean adults.

**Methods:**

We evaluated the 2018 Korean Community Health Survey data collected from community-dwelling people in Korea. A total of 104,453 non-smokers, 38,607 past smokers, and 26,776 current smokers with eligible data were included in the study. SCD was assessed using the Behavioral Risk Factor Surveillance System. The past or current smoking pack-years throughout each participant's entire life were calculated. Multiple regression analyses were carried out to estimate the adjusted odds ratios (ORs) as measures of the association between each type of smoking and SCD after adjustment for potential confounders.

**Results:**

Compared to no exposure, passive smoking was associated with higher odds of SCD. Compared to non-smokers, past smokers had a higher OR for SCD; however, current smokers did not. There were no significant associations between passive smoking and SCD in the non-smoker and past smoker groups, but there was a significant relationship between them in the current smoker group. While the cumulative dose of smoking was correlated with an increased OR of SCD in each group of current smokers and past smokers, E-cigarette smoking and the use of E-liquid were not associated with higher ORs in the current smoker group.

**Conclusion:**

Our findings support that passive smoking and past smoking are significantly associated with SCD and that more cumulative exposure to smoking is correlated with a higher OR of SCD.

## Introduction

Cigarette smoking is a widespread lifestyle practice with a prevalence of approximately 22.3% worldwide in 2020 (1). Although it is widely recognized that smoking is responsible for the development of a variety of chronic diseases, such as ischemic heart and respiratory disease, the prevalence of smoking remains high worldwide. Substantial evidence has demonstrated a role of nicotine in cognitive function. Several preclinical and clinical studies have demonstrated the cognitive benefit of acutely administered nicotine (2–4). Similarly, one meta-analysis revealed that nicotine could enhance cognitive function in multiple domains, including fine motor, alerting attention, orienting attention, short-term episodic memory, and working memory (5). However, a negative impact of smoking on cognition is becoming gradually accepted. Some observational studies indicated that compared to non-smoking, current smoking or cumulative cigarette exposure was associated with a 1.7- to 3.4-fold higher risk of amnestic mild cognitive impairment (MCI) or Alzheimer's disease (AD) in older adults (6–8). In addition, previous studies have reported that passive smoking can also increase the risk of cognitive impairment or dementia (9–11).

Subjective cognitive decline (SCD) is defined as a self-perception of a decline in cognitive performance without objective evidence of impairment on neuropsychological tests (12). Although numerous previous studies have reported that SCD is not related to progressive cognitive deterioration in most individuals, Slot et al. demonstrated that SCD could be an early indicator of future cognitive decline in some individuals (13). Given that most dementia, including AD, is irreversible and there is no current effective disease-modifying treatment for dementia, the importance of prevention is particularly emphasized. Therefore, investigating various factors related to SCD, which can be a pre-stage of dementia, may lead to an option for preventing subsequent dementia.

Although numerous studies have reported various risk factors for objective cognitive impairment, including smoking, there are limited studies on the factors associated with SCD. Furthermore, while previous studies have focused only on combustible cigarettes, few reports have investigated the relations between cognition and alternative nicotine/tobacco products that are becoming increasingly attractive to smokers, such as electronic nicotine delivery systems (E-cigarettes) or nicotine-containing e-liquid (E-liquid). Herein, we aimed to explore the relationship between current smoking/use and past smoking/use of combustible cigarettes, E-cigarettes and E-liquid, as well as passive smoking, and the incidence of SCD in Korean adults.

## Materials and Methods

### Study Population and Data Collection

This study was approved by the Institutional Review Board of the Korean Centers for Disease Control and Prevention (KCDC) (2016-10-01-T-A). Written informed consent was waived by the Institutional Review Board. All Korean Community Health Survey (KCHS) (14) data analyses were conducted according to the guidelines and regulations provided by the KCDC (Supplementary Material S1).

**Figure 1:**
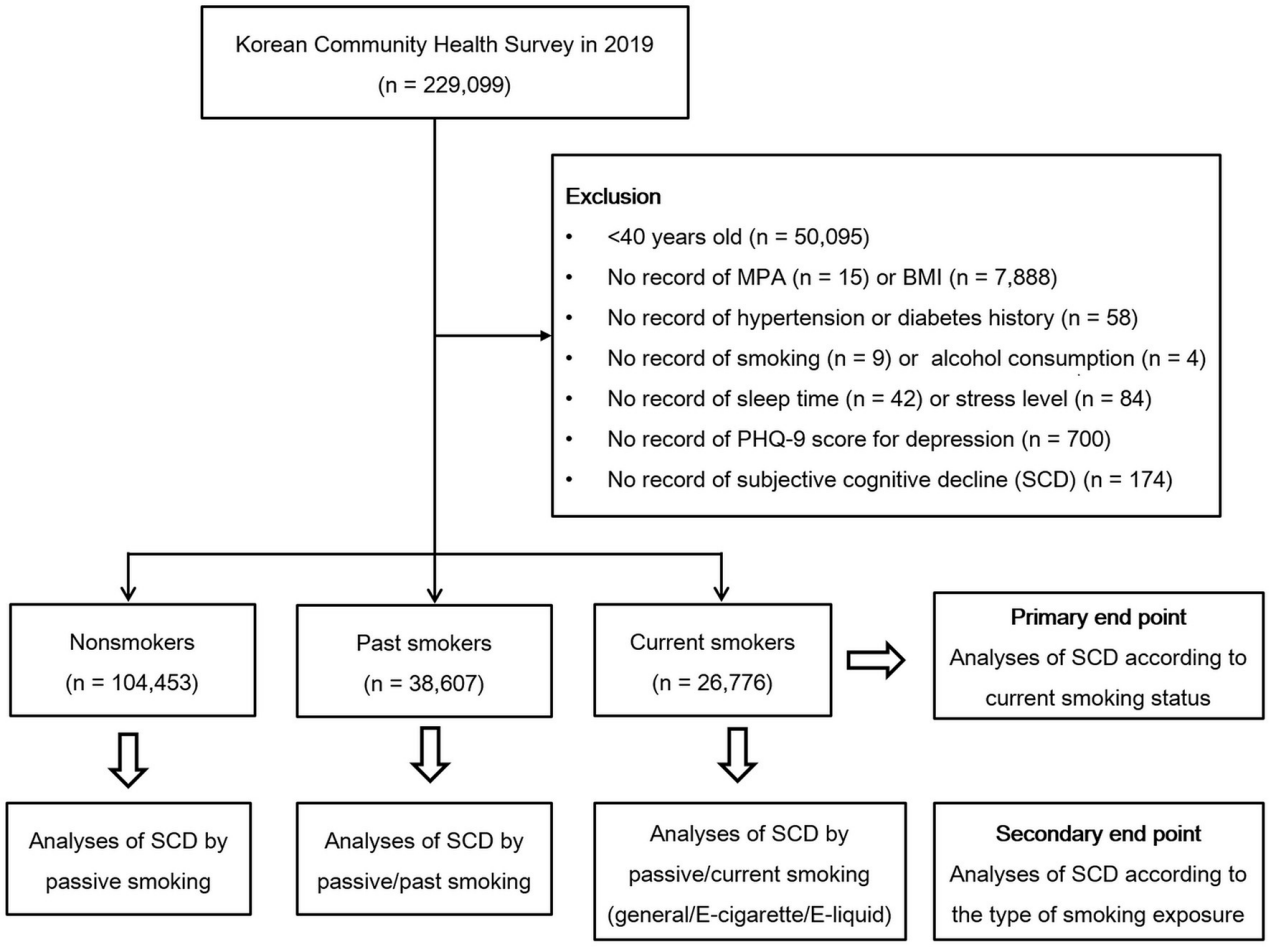
A schematic illustration of the participant selection process used in this study. A total of 104,453 nonsmokers, 38,607 past smokers, and 26,776 current smokers according to current smoking status were included in the analysis.

This study was a cross-sectional study using data obtained from the 2019 KCHS. The KCHS is an annual sample survey of 251 public health centers that began in 2008 and was conducted to understand the health status of local residents. These data are surveyed so that representative samples of adults aged 19 or older among the sample household members can be extracted. A total of 229,099 people participated in the 2019 KCHS, and trained investigators visited sample households in person and conducted measurement surveys and 1:1 interviews. A detailed description of the methods used for the selection of participants can be found in our previous studies (15, 16). Of the 229,099 total participants, we excluded participants from the present study for the following reasons: age under 40 years old (*n* = 50,095), no record of moderate-intensity physical activity (MPA) (*n* = 15), no record of body mass index (BMI) (*n* = 7,888), no record of hypertension or diabetes history (*n* = 58), no record of smoking (*n* = 9) or alcohol consumption (*n* = 4), no record of sleep time (*n* = 42) or subjective stress level (*n* = 84), no record of Patient Health Questionnaire (PHQ-9) (17) score for depression (*n* = 700), and no record of cognition survey (*n* = 174). The total number of participants included in the analysis was 169,836, and the participants were grouped into non-smokers (*n* = 104,453), past smokers (*n* = 38,607), and current smokers (*n* = 26,776) based on current smoking status (Figure 1). We analyzed the odds of SCD using the Behavioral Risk Factor Surveillance System (BRFSS) Questionnaire (CDC, 2008) (18) for cognitive decline according to current smoking status (primary end point). Additionally, we assessed the odds of SCD according to the type of smoking exposure in the non-smoker, past smoker, and current smoker groups (secondary end point).

### Questionnaire

#### Exposure

Smoking habits were surveyed using the questionnaire. Non-smokers were defined as participants who smoked fewer than 100 cigarettes throughout their life. Past smokers were defined as those who quit smoking. They were asked about the number of pack-years throughout their entire life. Current smokers were defined as those who were currently smoking. They were asked about general tobacco cigarette smoking (pack-years), current E-cigarette use (pack per day), and current E-liquid use within the previous month (yes/no). Passive smoking exposure (home or workplace) was surveyed in all three groups.

#### Outcomes

Participants who answered affirmatively to the question on the BRFSS (CDC, 2008) (18). “During the past 12 months, have you experienced confusion or memory loss that is happening more often or is getting worse?” were classified as having SCD. In other words, if the response was “Yes”, it was defined as SCD, and if the response was “No”, it was defined as not SCD. For SCD-related functional difficulties, cognitive decline in household activity, the need for assistance due to cognitive decline, and cognitive decline in social activity were assessed as follows: “During the last year, how often have your day-to-day activities (e.g., cooking, cleaning, taking medicine, driving, or paying bills, etc.) been hindered or needed help because of your confusion or memory loss?”; “If you needed help in daily life because you were confused or your memory was poor, how often did you receive help when you needed it?”; and “During the last year, how often have you been disturbed in your work life, volunteering, and social activities?” The response categories were always, usually, sometimes, rarely, or never.

#### Covariates

Information on factors that may be involved in cognitive function was collected. Age, sleep time (hours/day), and the PHQ-9 (17) score for depression (score from 0 to 27) were analyzed as continuous variables.

Sex, education level (middle school or lower, high school, and college or higher), MPA, obesity, hypertension and diabetes mellitus history, frequency of alcohol consumption (<1 time a month, 1–4 times a month, and ≥2 times a week), and subjective stress level (from very severe to no stress) were analyzed as categorical variables. MPA was calculated from the survey that collected information on the time spent participating in moderate- or high-intensity exercise each week (19), and MPA was categorized into three groups (0 min, 1–149 min, and ≥150 min). Obesity was assessed using BMI (kg/m^2^) and categorized as underweight (<18.5), normal (≥18.5 to <23), overweight (≥23 to <25), obese I (≥25 to <30), and obese II (≥30) (20). The detailed survey questionnaire is described on the KCDC website for KCHS (Prevention). The specified descriptions regarding exposures, outcomes, and covariates are represented in the Supplementary Table S2.

### Statistical Analysis

We calculated bivariate relationships between smoking status and participant characteristics using ANOVA for continuous variables and the chi-square test for categorical variables.

We estimated the crude and adjusted odds ratios (ORs) of passive smoking and current smoking status for SCD using multiple logistic regression with complex sampling by weighted values according to the recommendations of the KCHS to reflect the entire population of Korea. Age, sleep time, PHQ-9 score, sex, education level, MPA, obesity, subjective stress level, passive smoking, and current smoking status were included in the adjusted model.

To estimate the crude and adjusted ORs of various types of smoking (passive smoking, past smoking pack-years, current smoking pack-years, and E-cigarette and E-liquid use) for SCD in each current smoking status group (non-smoker, past smoker, and current smoker), multiple logistic regression with complex sampling was used. The same covariates used in the main models were included in the adjustment.

Additionally, adjusted ORs of various types of smoking (passive smoking, past smoking pack-years, current smoking pack-years, and E-cigarette and E-liquid use) for SCD-related functional difficulties in each current smoking status group were calculated using an ordinal logistic regression model.

We conducted subgroup analyses to verify possible effect modification by age (<60 years old, ≥60 years old) and sex. The cutoff point for age was determined according to the median value.

Two-tailed analyses were performed, and *p* values less than 0.05 were considered to indicate significance. The 95% confidence interval (CI) was also calculated. All ORs were estimated by taking into account the complex sampling structure of the data. All statistical analyses were conducted using SPSS v. 25.0 (IBM, Armonk, NY, USA).

## Results

Table 1 lists the general characteristics of the population comparing non-smokers, past smokers, and current smokers. The largest number of men was in the current smoker group (90.4%), while the number of women was highest in the non-smoker group (85.8%). For the alcohol drinking status, heavy drinkers were mostly distributed in the current smoker group (45.7%), but infrequent alcohol drinkers were primarily found in the non-smoker group 67.2%). The rates of passive smoking were 9.4, 12.2 and 20.5% among non-smokers, past smokers, and current smokers, respectively, with the highest in the current smoker group.

**Table 1:** General characteristics of the participants according to current smoking status.

**General characteristics**	**Number of participants**		
	**Non-smokers**	**Past smokers**	**Current smokers**
Total number	104,453	38,607	26,776
Age (years, mean, [SD])^*^	62.3 (12.6)	63.8 (12.1)	56.6 (11.0)
Sleep time (hour/day, mean, [SD])^*^	6.5 (1.3)	6.6 (1.3)	6.6 (1.3)
PHQ-9 for depression (score, mean, [SD])^*^	2.2 (3.1)	1.8 (2.8)	2.0 (3.1)
Sex, *n* (%)^†^			
Male	14,839 (14.2)	36,247 (93.1)	24,217 (90.4)
Female	89,614 (85.8)	2,360 (6.1)	2,559 (9.6)
Education level, *n* (%)^†^			
Middle school or lower	52,256 (50.0)	15,364 (39.8)	7,779 (29.1)
High school	29,534 (28.3)	12,481 (32.3)	11,291 (42.2)
College or higher	22,663 (21.7)	10,762 (27.9)	7,706 (28.8)
Moderate-intensity physical activity, *n* (%)^†^			
0 min	61,552 (58.9)	19,734 (51.1)	14,443 (53.9)
1–149 min	8,571 (8.2)	3,125 (8.1)	1,982 (7.4)
≥150 min	34,330 (32.9)	15,748 (40.8)	10,351 (38.7)
Obesity, *n* (%)^†^			
Underweight	3,403 (3.3)	1,016 (2.6)	1,086 (4.1)
Normal	38,916 (37.3)	11,134 (28.8)	9,558 (35.7)
Overweight	25,469 (24.4)	10,729 (27.8)	6,795 (25.4)
Obese I	31,468 (30.1)	14,093 (36.5)	8,174 (30.5)
Obese II	5,193 (5.0)	1,635 (4.2)	1,163 (4.3)
Hypertension history, *n* (%)^†^	37,568 (36.0)	15,643 (40.5)	7,290 (27.2)
Diabetes mellitus history, *n* (%)^†^	13,714 (13.1)	6,978 (18.1)	3,764 (14.1)
Frequency of alcohol drinking, n (%)^†^			
<1 time a month	70,189 (67.2)	15,742 (40.8)	7,611 (28.4)
1–4 time a month	24,477 (23.4)	10,110 (26.2)	6,930 (25.9)
≥2 times a week	9,787 (9.4)	12,755 (33.0)	12,235 (45.7)
Subjective stress level*, n* (%)^†^			
Very severe	2,442 (2.3)	716 (1.9)	1,002 (3.7)
Severe	17,948 (17.2)	5,744 (14.9)	5,917 (22.1)
A little	55,270 (52.9)	20,108 (52.1)	13,946 (52.1)
No	28,793 (27.6)	12,039 (31.2)	5,911 (22.1)
Passive smoking, *n* (%)^†^	9,861 (9.4)	4,720 (12.2)	5,487 (20.5)
SCD, *n* (%)^†^	23,117 (22.1)	7,837 (20.3)	4,222 (15.8)
SCD in household activity, *n* (%)^†^			
Never	92,268 (88.3)	34,734 (90.0)	24,855 (92.8)
Rarely	5,729 (5.5)	1,988 (5.1)	926 (3.5)
Sometimes	5,262 (5.0)	1,545 (4.0)	826 (3.1)
Usually	971 (0.9)	270 (0.7)	137 (0.5)
Always	223 (0.2)	70 (0.2)	32 (0.1)
Need of assistance due to SCD, *n* (%)^†^			
Never	94,224 (90.2)	35,326 (91.5)	25,177 (94.0)
Rarely	5,781 (5.5)	1,961 (5.1)	939 (3.5)
Sometimes	3,212 (3.1)	979 (2.5)	491 (1.8)
Usually	899 (0.9)	229 (0.6)	119 (0.4)
Always	337 (0.3)	112 (0.3)	50 (0.2)
SCD in social activity, *n* (%)^†^			
Never	95,387 (91.3)	35,636 (92.3)	25,318 (94.6)
Rarely	5,634 (5.4)	1,912 (5.0)	911 (3.4)
Sometimes	2,243 (2.1)	696 (1.8)	365 (1.4)
Usually	764 (0.7)	217 (0.6)	113 (0.4)
Always	425 (0.4)	146 (0.4)	69 (0.3)

*PHQ-9, Patient Health Questionnaire; SCD, subjective cognitive decline; SD, standard deviation*.

* *ANOVA*.

† *Chi-square test*.

This study showed that compared with no exposure, passive smoking was strongly associated with increased adjusted odds of SCD regardless of current smoking status (OR = 1.19, 95% CI = 1.13–1.26). Compared with non-smokers, past smokers also had higher odds of SCD independent of age or sex (OR = 1.19, 95% CI = 1.12–1.27). However, compared with not smoking, current smoking was not related to increased odds of SCD (OR = 1.00, 95% CI = 0.93–1.07, Table 2).

**Table 2:** Crude and adjusted odds ratios and 95% confidence intervals of passive smoking and current smoking status for subjective cognitive decline.

	**Odds ratios (95% confidence intervals)**	
	**for subjective cognitive decline**	
	**Crude**	**Adjusted^†^**
Total participants (*n =* 169,836)	
Passive smoking (reference = no exposure)	1.01 (0.95–1.05)	1.19 (1.13–1.26)^***^
Current smoking status (reference = non-smokers)	
Past smokers	0.87 (0.84–0.91)^***^	1.19 (1.12–1.27)^***^
Current smokers	0.67 (0.63–0.70)^***^	1.00 (0.93–1.07)
Age <60 years old (*n =* 76,800)
Passive smoking	1.24 (1.16–1.33)^***^	1.21 (1.12–1.30)^***^
Current smoking status	
Past smokers	0.82 (0.76–0.88)^***^	1.16 (1.04–1.29)^**^
Current smokers	0.77 (0.72–0.82)^***^	1.00 (0.90–1.11)
Age ≥60 years old (*n =* 93,036)
Passive smoking	1.01 (0.94–1.09)	1.14 (1.06–1.24)^**^
Current smoking status	
Past smokers	0.85 (0.81–0.90)^***^	1.18 (1.09–1.27)^***^
Current smokers	0.76 (0.70–0.81)^***^	1.02 (0.93–1.12)
Men (*n =* 75,303)
Passive smoking	1.02 (0.95–1.10)	1.24 (1.14–1.34)^***^
Current smoking status	
Past smokers	1.33 (1.24–1.43)^***^	1.17 (1.08–1.27)^***^
Current smokers	0.98 (0.90–1.06)	1.02 (0.93–1.11)
Women (*n =* 94,533)	
Passive smoking	1.10 (1.03–1.18 )^**^	1.17 (1.08–1.26)^***^
Current smoking status	
Past smokers	1.46 (1.29–1.65)^***^	1.18 (1.03–1.36)
Current smokers	1.27 (1.12–1.43)^***^	1.03 (0.90–1.19)

*Multiple logistic regression analysis with complex sampling*.

* *Significance at P < 0.05*.

** *Significance at P < 0.01*.

*** *Significance at P < 0.001*.

† *Adjusted for age, sleep time, Patient Health Questionnaire-9 score for depression, sex, education level, moderate-intensity physical activity, obesity, subjective stress level, passive smoking, and current smoking status*.

Table 3 presents a significant association between passive smoking and SCD in the non-smoker group (adjusted OR = 1.21, 95% CI = 1.12–1.30). This result was consistently significant in the subgroup analyses stratified by age and sex.

**Table 3:** Crude and adjusted odds ratios and 95% confidence intervals of passive smoking for subjective cognitive decline among non-smokers.

	**Odds ratios (95% confidence intervals)**	
	**for subjective cognitive decline**	
	**Crude**	**Adjusted^†^**
Total participants (*n =* 104,453)	
Passive smoking (reference = no exposure)	1.09 (1.02–1.17)^*^	1.21 (1.12–1.30)^***^
Age <60 years old (*n =* 45,565)	
Passive smoking	1.32 (1.20–1.45)^***^	1.22 (1.11–1.35)^***^
Age ≥60 years old (*n =* 58,888)	
Passive smoking	1.09 (0.99–1.21)	1.16 (1.05–1.29)^**^
Men (*n =* 14,839)	
Passive smoking	1.16 (0.96–1.40)	1.30 (1.07–1.59)^**^
Women (*n =* 89,614)	
Passive smoking	1.11 (1.03–1.19)^**^	1.19 (1.10–1.29)^***^

*Multiple logistic regression analysis with complex sampling*.

* *Significance at P < 0.05*.

** *Significance at P < 0.01*.

*** *Significance at P < 0.001*.

† *Adjusted for age, sleep time, Patient Health Questionnaire-9 score for depression, sex, education level, moderate-intensity physical activity, obesity, subjective stress level, passive smoking, and current smoking status*.

In the past smoker group, passive smoking and past smoking pack-years had adjusted ORs for SCD of 1.28 and 1.02, respectively (95% CI = 1.14–1.43 and 1.00–1.04, respectively). These increased ORs were maintained when the subjects were stratified by age and sex, except for past smoking pack-years in women and individuals ≥60 years. Rather, past smoking pack-years showed a lower adjusted OR for SCD in the subgroup of women (OR = 0.93, Table 4).

**Table 4:** Crude and adjusted odds ratios and 95% confidence intervals for subjective cognitive decline according to passive smoking and lifetime pack years among past smokers.

	**Odds ratios (95% confidence intervals)**	
	**for subjective cognitive decline**	
	**Crude**	**Adjusted^†^**
Total participants (*n =* 38,607)	
Passive smoking (reference = no exposure)	1.00 (0.90–1.11)	1.28 (1.14–1.43)^***^
Past smoking (10PYR)	1.10 (1.08–1.12)^***^	1.02 (1.00–1.04)^*^
Age <60 years old (*n =* 14,308)	
Passive smoking	1.39 (1.20–1.60)^***^	1.33 (1.14–1.54)^***^
Past smoking (10PYR)	1.09 (1.05–1.14)^***^	1.06 (1.01–1.11)^*^
Age ≥60 years old (*n =* 24,299)	
Passive smoking	0.98 (0.85–1.13)	1.19 (1.03–1.39)^*^
Past smoking (10PYR)	1.03 (1.01–1.05)^**^	1.01 (1.00–1.03)
Men (*n =* 36,247)	
Passive smoking	1.00 (0.89–1.11)	1.31 (1.17–1.48)^***^
Past smoking (10PYR)	1.12 (1.10–1.14)^***^	1.02 (1.00–1.04)^*^
Women (*n =* 2,360)	
Passive smoking	0.91 (0.73–1.13)	1.05 (0.83–1.31)
Past smoking (10PYR)	1.07 (1.01–1.13)^*^	0.93 (0.88–0.99)^*^

*PYR, pack-year. Multiple logistic regression analysis with complex sampling*.

* *Significance at P < 0.05*.

** *Significance at P < 0.01*.

*** *Significance at P < 0.001*.

† *Adjusted for age, sleep time, Patient Health Questionnaire-9 score for depression, sex, education level, moderate-intensity physical activity, obesity, subjective stress level, passive smoking, and current smoking status*.

The results in the current smoker group regarding the associations of SCD with passive smoking, current smoking pack-years, E-cigarette use, and E-liquid use are described in Table 5. Compared with no exposure, passive smoking was not significantly associated with higher odds of SCD in the current smoker group. More smoking pack-years were correlated with a higher OR of SCD, whereas E-cigarette or E-liquid use was not significantly associated with an increased OR for SCD in the current smoker group. Stratification by age and sex revealed that among women, more current smoking pack-years were associated with a higher OR for SCD and that E-cigarette use was associated with a lower OR for SCD.

**Table 5:** Crude and adjusted odds ratios and 95% confidence intervals for subjective cognitive decline according to passive smoking, E-cigarette, E-liquid use, and lifetime pack years among current smokers.

	**Odds ratios (95% confidence interval)**	
	**for subjective cognitive decline**	
	**Crude**	**Adjusted^†^**
Total participants (*n =* 26,776)	
Passive smoking (reference = no exposure)	1.06 (0.95–1.18)	1.08 (0.96–1.22)
Current smoking (10PYR)	1.09 (1.06–1.12)^***^	1.03 (1.00–1.06)^*^
E-cigarette (pack/day)	0.86 (0.61–1.21)	1.10 (0.77–1.59)
E-liquid use (reference = no use)	1.03 (0.84–1.28)	1.19 (0.93–1.51)
Age < 60 years old (*n =* 16,927)	
Passive smoking	1.19 (1.05–1.35)^**^	1.08 (0.94–1.24)
Current smoking (10PYR)	1.07 (1.03–1.12)^**^	1.04 (0.99–1.09)
E-cigarette (pack/day)	1.18 (0.86–1.63)	1.17 (0.83–1.67)
E-liquid use	1.21 (0.97–1.52)	1.18 (0.91–1.53)
Age ≥60 years old (*n =* 9,849)	
Passive smoking	1.00 (0.86–1.16)	1.06 (0.90–1.25)
Current smoking (10PYR)	1.00 (0.97–1.03)	1.02 (0.99–1.05)
E-cigarette (pack/day)	0.41 (0.10–1.58)	0.61 (0.17–2.19)
E-liquid use	1.30 (0.84–2.04)	1.27 (0.77–2.08)
Men (*n =* 24,217)	
Passive smoking	1.06 (0.94–1.19)	1.12 (0.98–1.26)
Current smoking (10PYR)	1.14 (1.11–1.17)^***^	1.02 (0.99–1.06)
E-cigarette (pack/day)	0.96 (0.68–1.36)	1.15 (0.79–1.66)
E-liquid use	1.09 (0.87–1.35)	1.20 (0.93–1.54)
Women (*n =* 2,559)	
Passive smoking	0.91 (0.73–1.13)	1.05 (0.83–1.31)
Current smoking (10PYR)	1.19 (1.11–1.28)^***^	1.13 (1.04–1.21)^*^
E-cigarette (pack/day)	0.36 (0.21–0.64)^***^	0.44 (0.22–0.88)^*^
E-liquid use	0.86 (0.58–1.27)	1.02 (0.67–1.56)

*PYR, pack-year. Multiple logistic regression analysis with complex sampling*.

* *Significance at P < 0.05*.

** *Significance at P < 0.01*.

*** *Significance at P < 0.001*.

† *Adjusted for age, sleep time, Patient Health Questionnaire-9 score for depression, sex, education level, moderate-intensity physical activity, obesity, subjective stress level, passive smoking, and current smoking status*.

The results of the relationship between passive smoking and past or current smoking pack-years with SCD-related functional difficulties in each current smoking status group are shown in Supplementary Tables S3–S5. Compared to no exposure, passive smoking was associated with lower SCD-related functional difficulties in past smokers. Past smoking pack-years were correlated with fewer cognitive decline in social activity among SCD-related functional difficulties (Supplementary Table S4).

## Discussion

This cross-sectional study assessed the association between the various types of smoking and the incidence of SCD among adults older than 40 years after adjustment for many covariates. Our results revealed that compared with non-smokers, current smokers had a lower possibility of SCD, whereas past smokers had a higher probability of SCD. The results of our study also showed that compared to no exposure, passive smoking was significantly related to SCD occurrence in the overall group of participants. This significant relationship between passive smoking and SCD was observed in non-smokers and past smokers but not in current smokers. Concerning the cumulative dose of smoking exposure, increased past smoking pack-years were related to SCD despite the inverse association in women. Similarly, current smoking pack years were associated with SCD among all current smokers, which seems to have been driven by the significant association in women. Regarding E-cigarette or E-liquid use, we found no significant relation to SCD.

Longitudinal studies have indicated a causal relationship between chronic smoking and an increased risk of cognitive decline and dementia. One meta-analysis that included prospective studies with at least 12 months of follow-up showed that elderly smokers in the general population are at a higher risk of cognitive decline than non-smokers (21). In a large cohort study, similar results were found for middle-aged smokers compared with non-smokers. Moreover, the risk of poor cognition was lower among people who had stopped smoking than among current smokers (22). The proposed mechanisms underlying the influence of smoking on cognitive function include endothelial damage, interference with brain oxygenation, increased oxidative stress, altered mitochondrial energy metabolism, decreased synaptic network connectivity, and metabolic enzymes related to amyloid beta or tau protein (23, 24). Although the negative effects and mechanism of smoking on cognitive performance have been increasingly established, our findings suggested that current smokers might be protected against SCD by enhancing cognitive function in the short term, which is consistent with a number of previous studies (2–4). This nicotine-induced enhancement of cognitive function may be explained by transient improvements in cognitive function via nicotinoid nerve excitation mediated by nicotine receptors (24).

Although a subgroup of women showed an inverse relationship in the past smoker group, this subgroup showed a significant result only in the current smoker group, and our overall results appear to indicate some relation between the number of pack-years and SCD in both the past smoker and current smoker groups. These findings could illustrate there was a meaningful relationship between cumulative exposure to chronic smoking and SCD, notwithstanding the transient compensation of current smoking for cognitive decline. However, the contradictory finding among women needs further investigation. In agreement with our analysis, several studies examining the link between pack-years and cognitive function showed that more pack-years correlated with a significantly higher rate of cognitive decline (25, 26). Another study investigating the effect of pack-years on changes in global cognitive function in elderly people without dementia found that more cigarette pack-years correlated with a significantly greater cognitive decline and that after a cutoff point of 10 pack-years, each additional pack-year was correlated with a 0.013-point decline in the Mini-Mental State Examination (MMSE) score (27).

Our findings revealed that passive smoking exposure had significant relationships with SCD, particularly among non-smokers, suggesting that passive smoking exposure could be more harmful to non-active smokers than to active smokers. Previous studies have shown the adverse effect of exposure to secondhand smoke on cognitive function in non-smokers (28, 29). Some studies have proposed that the possible effect of passive smoking on cognition may be due to a disrupted cholinergic system caused by nicotine and overstimulation of neurons implicated in learning and memory through long-term exposure (30, 31). Moreover, increased concentrations of carbon monoxide in the blood can impair oxygen flow to the brain, as occurs with air pollution (32).

Although E-cigarettes and other alternative nicotine and tobacco products have gradually become attractive to smokers, particularly young adults, very little is known about the safety and long-term health impact of these products. In particular, whether nicotine derived from E-cigarettes can impact cognitive processes has not been explored. One study investigating the effect of E-cigarettes on cognition showed that compared with placebo, the nicotine in E-cigarettes improved working memory performance, particularly at longer interference intervals. However, there was no effect of nicotine-containing E-cigarettes on letter cancelation performance when measuring attention and processing speed (33). Our study did not suggest a significant relationship between E-cigarette or E-liquid use and self-reported cognition. This might be the complex consequence of any possible effect of cigarette smoking cessation and the cumulative influence of long-term exposure to tobacco. Further research into cognition related to E-cigarette use should be conducted to determine a clear association.

Intriguingly, we found that passive smokers or past smokers with more pack-years had fewer SCD-related functional difficulties. A potential explanation is the possibility of reverse causality because of the cross-sectional nature of this study. That is, it is plausible that participants with more functional difficulties did not experience secondhand smoke or had fewer opportunities to have many smoking pack-years.

The main strength of this study is that we used data from a large, nationally representative sample of the Korean population, which allows us to increase generalizability. In addition, we adjusted for a variety of potential confounding factors, such as age, education level, sleep, physical activity, alcohol consumption, obesity, hypertension, diabetes, depression, and stress level, all of which can affect cognition, thus offering a more reliable estimate. A further strength of this study is that we classified the participants into three groups according to current smoking status and analyzed the impact of not only cigarette smoking but also E-cigarette smoking in each group, thereby providing a more specific association between different smoking types and SCD.

There are some limitations of this study. First, the design was cross-sectional, and the temporal effect of smoking on SCD remains unclear. This study could not conclude a causal relationship between the various smoking types and the incidence of SCD, since reverse causality cannot be ruled out. Future research, such as a longitudinal study, will be needed to clarify the causal association and temporal impact of smoking on SCD. Second, smoking and BRFSS data were self-reported and might be susceptible to recall and social desirability bias; thus, they are likely to have been underreported. Third, while we adjusted the models for a wide range of variables, there is still concern related to unmeasured confounding factors. Last, information regarding the use of E-cigarettes or E-liquids for non-smokers and past smokers has not been confirmed. Since many past smokers could quit smoking using E-cigarettes, they might have replaced combustible cigarettes. In addition, we also did not investigate the time of use or the concomitant use of combustible cigarettes and E-cigarettes.

## Conclusion

This study adds contemporary data to the growing body of evidence concerning the association between various types of smoking and self-reported cognitive decline. Specifically, more cumulative exposure to smoking was related to higher odds of SCD among both current smokers and past smokers, and passive smoking was also associated with SCD in both the smoker and non-smoker groups. Likewise, several studies highlighted that quitting smoking prior to age 40 eliminates all or most of the excess risk for a variety of diseases, while quitting later reduces the risks but does not eliminate them (34, 35). Despite the lack of causal relationships and the need for further investigations, these findings may support the need to stop smoking as soon as possible to prevent cognitive decline and subsequent dementia.

## Data Availability Statement

The data analyzed in this study was obtained from the Korea Community Health Survey (KCHS) database (http://KCHS.cdc.go.kr/), the following licenses/restrictions apply: KCHS controls access to this data and will grant access to any researcher who follows the established ethical approval process outlined by KCHS. Requests to access these datasets should be directed to KCHS, http://KCHS.cdc.go.kr/.

## Ethics Statement

The studies involving human participants were reviewed and approved by the Institutional Review Board of the Korean Centers for Disease Control and Prevention (KCDC; 2016-10-01-T-A). Written informed consent from the patients/participants' legal guardian/next of kin was not required to participate in this study in accordance with the national legislation and the institutional requirements.

## Author Contributions

JK and IC participated in the interpretation of the data and drafted and revised the manuscript. YK, CM, and DY participated in data collection and data interpretation. HC designed the study, participated in data collection and data interpretation, and revised the manuscript. All authors approved the final version of the manuscript for publication.

## Funding

This work was supported in part by a research grant from the National Research Foundation (NRF) of Korea (NRF-2021-R1C1C100498611 to HC and NRF-2018-R1C1B5083040 to JK).

## Conflict of Interest

The authors declare that the research was conducted in the absence of any commercial or financial relationships that could be construed as a potential conflict of interest.

## Publisher's Note

All claims expressed in this article are solely those of the authors and do not necessarily represent those of their affiliated organizations, or those of the publisher, the editors and the reviewers. Any product that may be evaluated in this article, or claim that may be made by its manufacturer, is not guaranteed or endorsed by the publisher.
