# Supplementary material for: Association Between Various Types or Statuses of Smoking and Subjective Cognitive Decline Based on a Community Health Survey of Korean Adults
Source: Front Neurol. 2022 Apr 29;13:810830. doi: 10.3389/fneur.2022.810830 (PMC9099047; doi:10.3389/fneur.2022.810830)
Supplement: Supplementary file 1 [file Table_1.DOCX]

**FILE S1** The description of the participant selection.

This study covers one nation using statistical methods based on designed sampling and adjusted weighted value. The KCHS conducted in 2019 were analyzed. The survey gathers information through face-to-face, paper assisted personal interviews between trained interviewers and respondents. The sample size for the KCHS is 900 subjects in each of 253 community units, including 16 metropolitan cities and provinces. The KCHS uses a two-stage sampling process clustered sampling. The first stage selects a sample area (tong/ban/ri) as a primary sample unit, which is selected according to the number of households in the area using a probability proportional to the sampling method. In the second stage, the number of households in the selected sample tong/ban/ri is identified to create a household directory. Sample households are selected using systematic sampling methods. This process is used to ensure that the sample units are representative of the entire population (Validity of self-reported healthcare utilization data in the Community Health Survey in Korea. *J Korean Med Sci* 26, 1409-1414). For the sample to be statistically representative of the population, the data collected from the survey were weighted by statisticians who performed post-stratification and considered the non-response rates (Prevalence and correlates of depressive symptoms in korean adults: results of a 2009 korean community health survey. *J Korean Med Sci* 28, 128-135)
