# Supplementary material for: Association Between Various Types or Statuses of Smoking and Subjective Cognitive Decline Based on a Community Health Survey of Korean Adults
Source: Front Neurol. 2022 Apr 29;13:810830. doi: 10.3389/fneur.2022.810830 (PMC9099047; doi:10.3389/fneur.2022.810830)
Supplement: Supplementary file 2 [file Table_2.docx]

**Table S2.** List of exposures, outcomes and covariates.

| **Questionnaire** | | **Definition or Assessment** | **Explanation** |
| --- | --- | --- | --- |
| **Exposure** | | | |
|  | Nonsmokers | Participants who smoked less than 100 cigarettes throughout their life |  |
|  | Past smokers | Participants who quit smoking | The number of pack-years during their entire life |
|  | Current smokers | Participants who were current  smoking | General tobacco cigarette smoking (pack-years)  Current E-cigarette use (pack per day)  Current E-liquid use within the previous month (yes/no) |
|  | Passive smoking | Home or workplace |  |
| **Outcome** | | | |
|  | Subjective cognitive decline (SCD)  SCD-related functional difficulties | Behavioral Risk Factor Surveillance System (BRFSS) questionnaire  BRFSS questionnaire | During the past 12 months, have you experienced confusion or memory loss that is happening more often or is getting worse?  During the last year, how often have your day-to-day activities (ex: cooking, cleaning, taking medicine, driving, or paying bills, etc.) been hindered or needed help because of your con- fusion or memory loss?; If you needed help in daily life because you were confused or your memory was poor, how often did you receive help when you needed it?; and During the last year, how often have you been disturbed in your work life, volunteering, and social activities? |
| **Covariates** | | | |
|  | Age |  |  |
|  | Sex |  |  |
|  | Sleep time | Hours/day |  |
|  | Patient Health Questionnaire (PHQ)-9 score for depression | The 9-item depression module from the full PHQ | 0 to 27 |
|  | Education level |  | Middle school or below, high school, college or over |
|  | Moderate-intensity physical activity (MPA) |  | 0 minutes, 1-149 minutes, ≥150 minutes |
|  | Obesity | Body mass index (BMI) | Underweight (<18.5), normal (≥18.5 to <23), overweight (≥23 to <25), obese I (≥25 to <30), and obese II (≥30) |
|  | Hypertension history |  |  |
|  | Diabetes mellitus history |  |  |
|  | Alcohol consumption |  | <1 time a month, 1-4 times a month, ≥2 times a week |
|  | Subjective stress level |  | Very severe, severe, a little, no stress |
