# Supplementary material for: Association Between Various Types or Statuses of Smoking and Subjective Cognitive Decline Based on a Community Health Survey of Korean Adults
Source: Front Neurol. 2022 Apr 29;13:810830. doi: 10.3389/fneur.2022.810830 (PMC9099047; doi:10.3389/fneur.2022.810830)
Supplement: Supplementary file 3 [file Table_3.docx]

**TABLE S3** Adjusted odds ratios and 95% confidence intervals of passive smoking for subjective cognitive decline-related functional difficulties in nonsmokers.

|  | **Cognitive decline in household activity** | | **Need of assistance due to cognitive decline** | | **Cognitive decline in social activity** | |
| --- | --- | --- | --- | --- | --- | --- |
|  | Adjusted odds ratio^†^  (95% confidence interval) | *P* value | Adjusted odds ratio^†^  (95% confidence interval) | *P* value | Adjusted odds ratio^†^  (95% confidence interval) | *P* value |
| Passive smoking (reference = no exposure) | 0.95 (0.86-1.06) | 0.355 | 0.95 (0.84-1.07) | 0.374 | 0.92 (0.81-1.04) | 0.191 |

Ordinal logistic regression analysis with complex sampling.

^†^Adjusted for age, sleep time, Patient Health Questionnaire-9 score for depression, sex, education level, moderate-intensity physical activity, obesity, subjective stress level, passive smoking, and current smoking status.
