# Supplementary material for: Association Between Various Types or Statuses of Smoking and Subjective Cognitive Decline Based on a Community Health Survey of Korean Adults
Source: Front Neurol. 2022 Apr 29;13:810830. doi: 10.3389/fneur.2022.810830 (PMC9099047; doi:10.3389/fneur.2022.810830)
Supplement: Supplementary file 4 [file Table_4.docx]

**TABLE S4** Adjusted odds ratios and 95% confidence intervals for subjective cognitive decline-related functional difficulties according to passive smoking and lifetime pack years among past smokers.

|  | **Cognitive decline in household activity** | | **Need of assistance due to cognitive decline** | | **Cognitive decline in social activity** | |
| --- | --- | --- | --- | --- | --- | --- |
|  | Adjusted odds ratio^†^  (95% confidence interval) | *P* value | Adjusted odds ratio^†^  (95% confidence interval) | *P* value | Adjusted odds ratio^†^  (95% confidence interval) | *P* value |
| Passive smoking (reference = no exposure) | 0.83 (0.71-0.98) | 0.025^*^ | 0.81 (0.68-0.98) | 0.026^*^ | 0.77 (0.64-0.93) | 0.006^*^ |
| Past smoking (10PYR) | 0.97 (0.95-1.00) | 0.056 | 0.98 (0.96-1.00) | 0.054 | 0.97 (0.95-0.99) | 0.011^*^ |

*PYR, pack-year.*

Ordinal logistic regression analysis with complex sampling.

^†^Adjusted for age, sleep time, Patient Health Questionnaire-9 score for depression, sex, education level, moderate-intensity physical activity, obesity, subjective stress level, passive smoking, and current smoking status.
