# Supplementary material for: Association Between Various Types or Statuses of Smoking and Subjective Cognitive Decline Based on a Community Health Survey of Korean Adults
Source: Front Neurol. 2022 Apr 29;13:810830. doi: 10.3389/fneur.2022.810830 (PMC9099047; doi:10.3389/fneur.2022.810830)
Supplement: Supplementary file 5 [file Table_5.docx]

**TABLE S5** Adjusted odds ratios and 95% confidence intervals for subjective cognitive decline-related functional difficulties according to passive smoking, E-cigarette, E-liquid use, and lifetime pack years among current smokers.

|  | **Cognitive decline in household activity** | | **Need of assistance due to cognitive decline** | | **Cognitive decline in social activity** | |
| --- | --- | --- | --- | --- | --- | --- |
|  | Adjusted odds ratio^†^  (95% confidence interval) | *P* value | Adjusted odds ratio^†^  (95% confidence interval) | *P* value | Adjusted odds ratio^†^  (95% confidence interval) | *P* value |
| Passive smoking (reference = no exposure) | 0.90 (0.76-1.06) | 0.209 | 0.98 (0.81-1.19) | 0.854 | 0.94 (0.78-1.13) | 0.504 |
| Current smoking (10PYR) | 1.01 (0.97-1.06) | 0.597 | 1.01 (0.96-1.05) | 0.767 | 1.01 (0.96-1.06) | 0.663 |
| E-cigarette (pack/day) | 0.90 (0.51-1.59) | 0.715 | 1.16 (0.59-2.32) | 0.665 | 1.00 (0.52-1.95) | 0.992 |
| E-liquid use (reference = no use) | 0.85 (0.57-1.27) | 0.437 | 0.78 (0.51-1.19) | 0.243 | 0.74 (0.48-1.14) | 0.167 |

*PYR, pack-year.*

Ordinal logistic regression analysis with complex sampling.

^†^Adjusted for age, sleep time, Patient Health Questionnaire-9 score for depression, sex, education level, moderate-intensity physical activity, obesity, subjective stress level, passive smoking, and current smoking status.
